# En Bloc Resection and Pelvic Ring Reconstruction for Primary Malignant Bone Tumors Involving Sacroiliac Joint

**DOI:** 10.1111/os.12563

**Published:** 2019-11-22

**Authors:** Ming Xu, Kai Zheng, Jie Zhao, Wen‐zhe Bai, Xiu‐chun Yu

**Affiliations:** ^1^ Department of Orthopaedics The 960th Hospital of PLA Jinan China

**Keywords:** Primary malignant bone tumors, Sacroiliac articulation, Transarticular invasion, Bone Cancer, Sacroiliac Joint, Resection Margin

## Abstract

**Objective:**

To observe the process of sacroiliac joint invasion by primary malignant tumors of sacrum and iliac bone, and to explore the methods of surgical resection and reconstruction.

**Methods:**

From January 2009 to November 2017, there were nine patients with primary malignant bone tumors involving sacroiliac joints, five males and four females, aged from 16 to 63 years, with an average age of 35 years. Of these there were three cases of primitive neuroectodermal tumors, three cases of chondrosarcoma, and three cases of osteosarcoma. Pelvic ring reconstruction was performed with longitudinal half sacrum, sacroiliac joint and partial iliac bone block excision and screw‐rod system combined with bone grafting.

**Results:**

The operation time was 155–310 min, with an average of 245 ± 55 min, and the bleeding volume was 1400–8500 ml, with an average of 3111 ± 2189 ml. Follow‐up ranged from 5 to 108 months, with a median follow‐up of 24 months. Three patients (33.3%) had local recurrence, three patients (33.3%) survived without tumors, and one patient had lung metastasis 2 years after operation, and survived with tumors. Five patients (55.6%) died, of which four died of lung metastasis and one died of brain metastasis. Survival analysis showed that the 3‐year overall survival rate was 57%. Bone grafts did not heal in four patients, and bone grafts healed in five patients. The healing time ranged from 5 to 7 months, with an average of 6.2 months. Complications: one patient developed deep infection 2 months after operation; one patient had skin edge necrosis; titanium rod loosening and displacement were found in two patients with nonunion of bone graft, and no fracture of nail rod was found. The MSTS 93 functional score of nine patients ranged from 20% to 50%, with an average of 34%.

**Conclusion:**

The tumors around the sacroiliac joint often invade the contralateral bone by ligament, and the en bloc resection and pelvic ring reconstruction for primary malignant bone tumors involving sacroiliac joint was feasible.

## Introduction

Malignant bone tumors of the ilium or sacrum adjacent to the sacroiliac joint may invade the sacroiliac joint to the contralateral bone. Ozaki *et al*.^1^ analyzed 51 cases of primary malignant iliac tumors invading sacrum adjacent to sacroiliac joint (less than 2 cm away from sacroiliac joint). Postoperative pathology confirmed that 15 cases invaded sacroiliac joint through sacroiliac joint, the incidence was 29.4%. Chhaya *et al*.^2^ confirmed by imaging and histology after operation that in 12 out of 24 patients with malignant tumors of adjacent sacroiliac joints that were invaded, the incidence was 50%. Ding*et al*.^3^ analyzed magnetic resonance imaging (MRI) data of 93 patients with primary malignant bone tumors of the ilium and sacrum adjacent to the sacroiliac joint. Postoperative pathology confirmed 27 patients (29.0%) with transsacroiliac joint invasion.

For primary malignant bone tumors involving the sacroiliac joint, it is necessary to resect the ilium and sacrum invaded by the tumors together with the sacroiliac joint. Because the sacroiliac joint is adjacent to the lumbosacral nerve, iliac vessels, rectum, bladder, and other important tissues and organs, it is difficult to achieve extensive resection of the malignant tumors in this area. It is difficult to select and expose the surgical approach. The difficulties faced by the whole resection of tumors include: how to expose the tumor; how to resect to reach the safe boundary of oncology; how to avoid intraoperative nerve and organ injury; and how to avoid intraoperative hemorrhage and other complications. Court *et al*.^4^ treated 40 cases of malignant tumors involving sacroiliac joint. The recurrence rate was 7% in patients reaching the safe boundary and 70% in patients not reaching the safe boundary. Yang *et al*.^5^ performed limb salvage surgery on 60 cases of pelvic malignant tumors involving sacrum. Thirty‐three cases failed to reach the safe surgical boundary, the recurrence rate was 51.6%, 27 cases reached the edge or extensive resection boundary, and the recurrence rate dropped to 22.2%. It is more difficult to reconstruct pelvic ring integrity after resection of tumors. Beadel^6^ and Niu^7^ advocated that the pelvic ring should not be reconstructed after resection of iliosacral joint tumors. More and more literature reported different methods of pelvic ring reconstruction^4^, ^8^, ^9^, ^10^, ^11^, which can be divided into two types: one is screw rod internal fixation combined with bone grafting, the other is titanium plate and screw internal fixation combined with bone grafting.

We retrospectively analyzed the clinical data of nine patients with primary malignant bone tumors involving sacroiliac joint, who underwent longitudinal hemi‐sacral resection with sacroiliac joint block resection and pelvic ring reconstruction with screw‐rod system combined with bone grafting. The purpose was: (i) to observe the way in which primary malignant tumors of sacroiliac joint invaded sacroiliac joint; (ii) to explore the surgical approach and resection margin; and (iii) to summarize the results of the reconstruction of sacroiliac joint stability.

## Materials and Methods

### *Database of Patients*

Inclusion criteria: (i) primary malignant bone tumors involving sacroiliac joint; (ii) discontinuous pelvic ring after surgical resection; and (iii) marginal resection of surgical margin. Exclusion criteria: (i) metastatic tumors; (ii) aggressive tumors, such as giant cell tumors of bone; (iii) tumors that invade acetabulum or obturator area; and (iv) tumors that invade lumbar vertebrae or sacrum. According to inclusion and exclusion criteria, from January 2009 to November 2017, nine patients with primary malignant bone tumors involving sacroiliac joints were treated in our department, including five males and four females, aged 16–63 years, with an average age of 35 years. Primitive neuroectodermal tumors were found in three cases, chondrosarcoma in three cases, and osteosarcoma in three cases. Except one case of recurrent chondrosarcoma that did not undergo preoperative biopsy, the other eight cases underwent preoperative puncture biopsy to make a definite diagnosis.

### *Preoperative Preparation and Operative Plan*

Preoperative diagnosis of osteosarcoma and primitive neuroectodermal tumors were treated with cisplatin combined with adriamycin combined with isocyclophosphamide for two courses. Each patient's surgical plan was made according to the preoperative imaging. The surgical resection range included sacrum combined with sacroiliac joint combined with iliac bone.

### *Surgical Methods*

The surgical methods are as follows.

#### *Tumor margin exposure*

The patient took lateral decubitus position and the affected side was on the upper side. First, the posterior median longitudinal incision of the sacrum was taken to open the paravertebral muscles. At the sacral level, the sacral lamina and spinous processes were exposed and sacral tubercle ligaments and sacrospinal ligaments were incised. Then, the ilioinguinal incision was taken to extend posteriorly along the iliac crest to the median sacral incision, and the gluteus maximus muscle was separated from the ischial foramen at the safe boundary of the lateral pelvic tumors. During the anatomical period, the superior gluteal artery was preserved as far as possible; the three layers of muscles of the abdominal wall were cut in the medial pelvis, and the iliac muscle was cut or separated from the medial iliac bone to protect the iliac vessels, ureters, and abdominal organs, and expose the ante The medial sciatic foramen protects the sciatic nerve, and expose the anterior sacroiliac joint and the medial sciatic foramen to protect the sciatic nerve and the lumbar quadratus muscle and iliolumbar ligament are cut along the posterior iliac crest to expose the upper part of the sacral wing.

#### *Extramarginal osteotomy of tumors*

**Figure 1:**
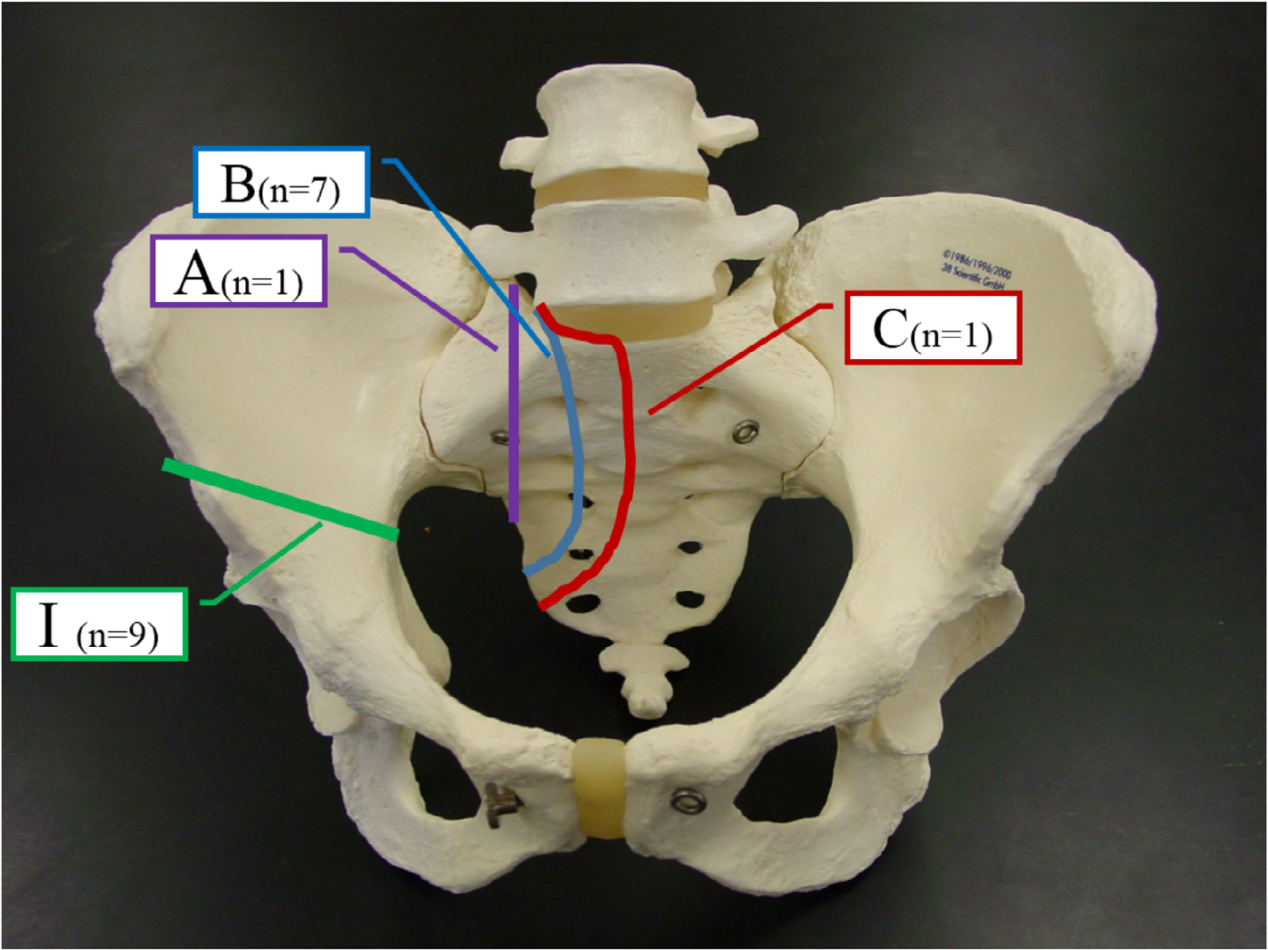
The types and cases of iliac and sacral osteotomy in nine patients in this group. Iliac osteotomy: type I, osteotomy above acetabulum, nine cases; sacral resection: type A, lateral sacral foramen osteotomy, one case; type B, sacral foramen osteotomy, seven cases; type C, median sacral osteotomy, one case.

Court *et al*.^4^ divided sacral resection into four types (A, B, C, and D) and pelvic resection into four types I, II, IIp, III (Fig. 1). According to preoperative images, iliac osteotomy was performed on the top of acetabulum in nine patients, which was type I. Sacral resection can be classified into three types. Type A, where the tumors only invade part of the sacral wing and osteotomy is performed lateral to the sacral foramen. There was one case in this group. Type B, where the tumors did not invade the sacral foramen, or the lump was located in front of the sacral foramen. Osteotomy was performed at the sacral foramen. If the tumors invaded the lumbosacral nerve, they could be removed together. There were seven cases in this group, including one case with L_5_ nerve cut off, one case with L_5_ and S_1_ nerve cut off. Type C, where the tumors invaded part of sacrum, exposed sacral canal and dural sac during operation, ligated one side of sacral nerve, osteotomy along sacral axis, resected part of L_5_S_1_ intervertebral disc, and sacral hemisection. There was one case in this group. The affected side of L_5_, S_1_, and S_2_ nerve roots were cut off. After iliac and sacral osteotomy, the tumors were removed completely to stop bleeding.

#### *Reconstruction*

Pelvic rings were reconstructed with screw‐rod system after resection of tumors. In L_5_, pedicle screw was implanted through pedicle. In S_1_, pedicle screw was implanted into anterolateral sacrum through pelvic approach. In the remaining iliac bone, two pedicle screws were implanted along the direction of ischium and pubis. Titanium rod was placed and top screw was tightened. All patients underwent autologous bone transplantation between sacrum and iliac bone, eight patients underwent autologous iliac bone transplantation, and one patient underwent autologous fibular bone transplantation. Titanium mesh with bone grafting was carried out in two patients.

### *Postoperative Management*

Six weeks after the operation, part of the patients using crutches were walking with load, and 3 months after the operation, all the patients using crutches were walking with load. Adjuvant chemotherapy were administered after incision healing in patients with primitive neuroectodermal tumors and osteosarcoma.

### *Oncological Outcome*

Survival status was evaluated according to both local and distant tumor control. The patients were evaluated at 3‐month intervals by chest computed tomography (CT) and X‐rays of the operative site. Clinical and radiological assessments were performed at each visit to determine local recurrence or metastasis. Follow‐up was conducted once every 3 months in the first year, once every 6 months in the second year, and once a year thereafter. Outpatient follow‐up was the main method. The follow‐up included physical examination and imaging examination.

### *Healing of Bone Graft*

The bone graft healing results were evaluated at 3‐month intervals by CT and X‐rays of the operative site. There are three kinds of cases: (i) bone union, continuous bone trabeculae run through the whole bone graft site; (ii) bone nonunion, bone graft area showed a low density area of bone absorption, with a circular sclerosing zone around it; and (iii) partial bone union. Some continuous and orderly bone trabeculae penetrate the bone graft site, some regions are union, some regions are nonunion as small or scattered low density areas, and there are sclerotic zones around them.

### *Functional Outcome*

Functional outcomes were evaluated using the Musculoskeletal Tumor Society (MSTS) 1993 scoring system^12^ for the lower extremity. The final MSTS score is calculated as a percentage of the maximum possible score; the higher the percentage, the better the functional outcome. Functional scores were measured at 3, 12, and 24 months postoperatively.

### *Complications*

The clinical complications, such as infection, incision complications, and titanium rod loosening, were recorded. Oncological failure was not recorded as a complication. The muscle strength of tibialis anterior muscle, extensor pollicis longus muscle, and triceps calf muscle was evaluated by muscle strength grading method (0–5 grade).

### *Statistical Method*

SPSS 20.0 (SPSS Company, USA) was used for statistical analysis. Metrological data were expressed by mean ± standard deviation and survival analysis was performed by Kaplan‐Merier method.

## Results

### *Oncological Outcome*

**Figure 2:**
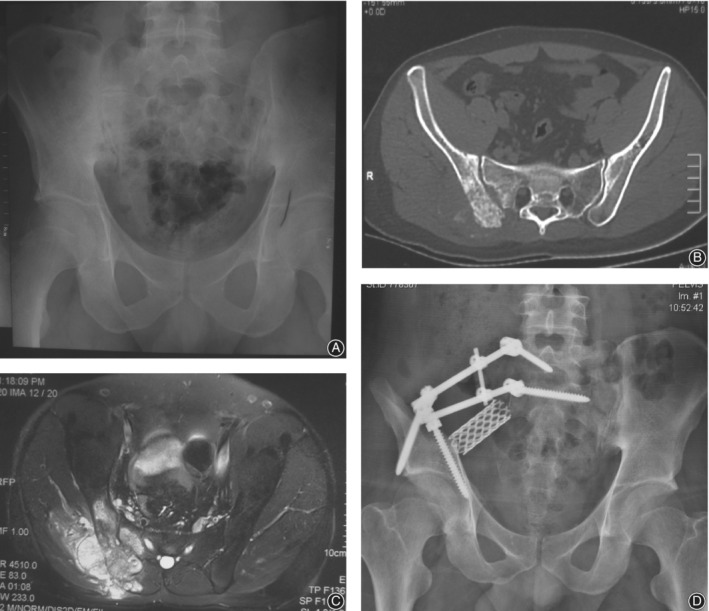
Male, 27 years old, right iliac sacral osteosarcoma. (A) Positive pelvic X‐ray showed sclerotic bone destruction of the right posterior iliac and sacrum before operation. (B) Preoperative CT showed bone destruction and soft tissue mass formation in the posterior part of the right iliac bone, which invaded the right sacral wing. (C) Preoperative MRI showed that the tumors invaded the sacroiliac joint and the right sacral wing through the interosseous ligament. Soft tissue mass invaded the gluteus maximus and gluteus medius muscle. (D) Positive pelvic X‐ray films 1.5 years after operation showed good internal fixation and titanium mesh position without signs of loosening or rupture.

Nine patients were followed up for 5 to 108 months, with a median follow‐up time of 24 months. Three cases (33.3%) had local recurrence, including one case of chondrosarcoma recurred half a year after operation, two cases of osteosarcoma recurred 1.5 years and 3 years after operation, three cases (33.3%) had no tumor survival (Fig. 2), one case had lung metastasis 2 years after operation, and the present survived with tumor. One case of lung metastasis are alive with neoplasm five patients (55.6%) died, of which four died of lung metastasis and one died of brain metastasis. Survival analysis showed that the 3‐year overall survival rate was 57% (20.1% of the cumulative survival rate standard error).

### *Healing of Bone Graft*

Bone grafts did not heal in four patients, of which two patients had bone graft resorption, one patient died of brain metastasis 5 months after operation, and one patient had deep infection and bone graft resorption. The healing time of bone graft was 5 to 7 months (average, 6.2 months).

### *Functional Outcome*

The average functional results of living patients was 34% (range, 20%–50%) according to MSTS 1993. One patient underwent resection of the L_5_ and S_1_ nerves on the affected side during the operation, resulting in postoperative tibialis anterior muscle strength grade 3, extensor pollicis longus muscle strength grade 0, triceps calf muscle strength grade 3; L_5_ nerve of affected side was cut off during operation in one case, and muscle strength of extensor pollicis longus was 0 grade after operation; and one patient underwent resection of the S_1_‐S_3_ nerves on the affected side, resulting in muscle strength of triceps surae grade 2 after operation.

### *Complications*

Deep infection occurred in one case, 2 months after operation. Debridement and suture were given, long‐term exudation was performed, with recurrence occurring half a year after operation, and pulmonary metastasis 11 months after operation and pulmonary metastasis occurred 11 months after operation. One patient had skin edge necrosis and healed after debridement and suture. Titanium rod loosening and displacement occurred in two patients with nonunion of bone graft at 11 months and 24 months, respectively. No fracture of nail rod occurred.

## Discussion

### *Incidence and Manner of Sacroiliac Joint Invasion by Malignant Sacroiliac Tumors*

Primary malignant bone tumors adjacent to the sacroiliac joints have a deeper location, and may invade the sacroiliac joints or even the transsacroiliac joints at the time of consultation. Preoperative determination of whether there is trans‐articular invasion of sacroiliac and iliac tumors has guiding significance for the formulation of surgical treatment. Chhaya *et al*.^2^ observed nine sacroiliac joint specimens by gross anatomy, and pointed out that the transarticular invasion of bone tumors adjacent to sacroiliac joint included: (i) transarticular invasion along the posterior ligament of sacroiliac joint (transarticular invasion through ligament connection); (ii) direct destruction of the anterior and inferior cartilage of sacroiliac joint and intraarticular transarticular invasion (transarticular cartilage invasion through articular cartilage); and (iii) trans‐articular invasion spreads along the growth of soft tissues, such as muscles and ligaments around the sacroiliac joint (peri‐articular invasion). Tumors may coexist in two or more trans‐articular invasion modes. It was confirmed by imaging and histology after operation that 12 of 24 patients with malignant tumors adjacent to sacroiliac joint were invaded, eight of them had invaded sacroiliac joint by ligament connection. Twelve of the twenty‐four patients demonstrated imaging and histological evidence of transarticular sacroiliacjoint invasion. Eight tumours infiltrated only the interosseous ligamentousaspect of the sacroiliac joint. In the other four cases, giant tumors directly invaded articular cartilage and ligament, and no case involved articular cartilage alone. Ding *et al*.^3^ analyzed MRI data of 93 patients with primary malignant bone tumors of ilium and sacrum adjacent to sacroiliac joint confirmed by operation and pathology. Twenty‐seven patients with transsacroiliac joint invasion were found. It is concluded that MRI is highly sensitive for showing transarticular invasion of bone tumors adjacent to the sacroiliac joint. There was no significant difference in the incidence of transsacroiliac joint invasion between iliac and sacroiliac primary malignant bone tumors adjacent to the sacroiliac joint. The incidence of transsacroiliac joint invasion of common osteosarcoma and Ewing sarcoma was significantly higher than that of central chondrosarcoma and chordoma with lower malignant degree. Bone tumors often invade the contralateral bone by ligament connection across sacroiliac joint. Among the nine cases, eight cases were iliac malignant tumors invading sacrum and one case was sacral malignant tumors invading iliac bone. Among them, six cases invaded contralateral bone by ligament connection and three cases invaded contralateral bone around transsacroiliac joint because of huge tumors.

### *Surgical Approach and Tumor Resection Margin*

It is very difficult for malignant tumors involving the sacroiliac joint to reach a safe surgical boundary. A reasonable surgical incision is conducive to the complete removal of the tumors. For malignant sacroiliac joint tumors that invade part of the sacrum, we adopt a single, lateral swaying position, a posterior median sacral approach combined with an ilioinguinal approach, which can better expose and resect the tumors. Through the posterior median sacral approach, the posterior lamina and sacral foramen of the sacrum can be exposed, the ligaments around the sacrum can be cut off, the dura mater of the spinal canal can be exposed, and the sacral nerve can be cut off on the affected side when necessary. Through the ilioinguinal approach, pelvic vessels and nerves can be protected, and the structure of the sciatic foramen, the superior and anterior sacroiliac joints can be exposed, which is conducive to tumor resection. Wang and Sabourin^8^, ^11^ used this approach for surgery.

Anatomically, resection of malignant tumors of the iliosacral joint involves sacroiliac joints and surrounding muscles and nerves. It is difficult to obtain a safe margin of resection because most tumors are large, adjacent to important nerve and vascular structures, and involve the invasion of tumors of the sacrum and sacral nerve roots. The quality of resection margin is closely related to the recurrence rate. From 1982 to 2001, Court *et al*.^4^ examined 40 cases of tumors involving resected sacroiliac joint. 74% of the patients reached the safe margin, 26% of the patients did not reach the safe margin. The recurrence rate was 7% among the patients who reached the safe margin, and 70% of the patients who did not reach the safe margin relapsed. From 1999 to 2011, Yang *et al*.^5^ performed limb salvage surgery on 60 cases of pelvic malignant tumors involving the sacrum. Thirty‐three cases failed to reach the safe surgical boundary. Of them, 17 cases (51.6%) had local recurrence 3–18 months after operation, 27 cases reached the margin or extensive resection boundary, and only six cases (22.2%) had local recurrence. Laitinen *et al*.^13^ applied navigation technology to resect malignant sacroiliac joint tumors. There were nine cases in the navigation group, including two cases with local recurrence (22.2%), and 12 cases in a non‐navigation group, six cases with recurrence (50.0%). The authors believe that navigation‐assisted surgery for resection of posterior sacroiliac tumors increases patient safety and provides better oncological outcomes. Marginal resection was performed in all nine patients. Local recurrence occurred in three patients (33.3%) after operation. The results were similar to those in the literature.

### *Reconstruction of Sacroiliac Joint Stability*

Two scholars advocated that pelvic ring should not be reconstructed after resection of iliosacral joint tumors. Beadel *et al*.^6^ underwent resection of sacroiliac joint tumors in 16 patients, 12 of whom had not reconstructed pelvic ring and four had reconstructed pelvic ring. There was no difference in MSTS and TESS scores between the two groups, but the patients without reconstruction had shorter operation time, less bleeding, fewer operative complications, and the same local and systemic tumor control. The functional results were similar or better than those of the patients with bone defect. The authors believe that there is a biomechanical advantage of not reconstructing the iliosacral joint after resection. When not reconstructed, the medialization of the hip closer to the center of gravity means that the moment generated by weight is smaller and can act on the shorter lever arm, which leads to the improvement of the patient's one‐leg posture. When no reconstruction is done, medialization of the hip joint closer to the center of gravity means the moment produced by the body weight is smaller because it acts over a shorter lever arm, which results in improved single‐leg stance for the patient. Niu *et al*.^7^ performed resection of sacroiliac joint tumors in 21 patients, 18 of whom were not reconstructed, and three of whom were reconstructed. There was no difference in limb shortening length between the two groups, and there was no significant difference in MSTS and TESS scores between the two groups. The authors suggest that iliosacral resection without reconstruction may be a feasible treatment option for pelvic I  and IV tumors. However, there were also opposite results. Shen *et al*.^8^ treated 26 cases of malignant sacroiliac joint tumors, including 12 cases of fibula transplantation plus reconstruction of plate or pedicle screw, 14 cases of which were not reconstructed. The MSTS score of reconstruction group was 25.25 ± 2.93, and that of non‐reconstruction group was 20.36 ± 2.56. The functional score of bio‐reconstruction group was significantly higher than that of non‐reconstruction group.

More papers reported different methods of pelvic ring reconstruction, mainly divided in two ways: one is screw rod internal fixation and bone grafting, the other is titanium plate screw internal fixation combined with bone grafting. In the literature of internal fixation with screw rods, Nassif *et al*.^9^ used two nails and one rod. Two pedicle screw were screwed into the iliac bone and sacrum, connected with the rod, and reconstructed the pelvic ring after bone grafting. However, Louer *et al*.^14^ found that the displacement of sacrum and iliac bone increased more easily when loaded vertically with two nails and one rod. Court^4^ and Ye^10^ were fixed with four screws and two rods, two pedicle screws were fixed with iliac bone, one pedicle screw was fixed with lumbar five L5 and sacrum respectively, and two rods were connected. Ye *et al*.^10^ performed pelvic I and  IV resection and reconstruction in six patients. The space of the screw in the sacrum was limited. It was difficult to screw two screws into the sacrum. One screw was fixed on the sacrum and the other was fixed on L_5_. Compared with pedicle screw, lateral lumbar/sacral screw is recommended because of its biomechanical advantages. Of the nine patients we treated, eight were fixed with four screws and two rods, of which six were fixed with two screws in L_5_ and S_1_ respectively, and the other two were fixed with two screws in sacrum. One patient was fixed with two nails and one rod. Sabourin *et al*.^11^ pointed out that in the case of sacral hemisection, pedicle screw should be fixed to L_5_ vertebral body, and the healthy side L_5_‐S_1_ also need to be implanted with a pedicle screw. The author advocates that autologous iliac bone transplantation should be the first choice. The author advocated that autologous iliac bone transplantation should be the first choice. The overall healing rate of bone graft is 58%. Radiotherapy and chemotherapy have significant effects on the healing of bone graft. Wang *et al*.^8^ fixed six patients with AO reconstruction plate, four patients with four nails and two rods, and took autologous double row fibula or allograft bone. Of the 12 patients, 11 (91%) had bone union, with an average time of 11 months (8–14 months). The authors believe that non‐vascularized fibula is simpler, cheaper, and faster. Nassif *et al*.^9^ used iliac bone graft with medial gluteal muscle pedicle on the affected side and single screw rod fixation of sacroiliac bone in six patients. One case had bone healing after operation, and four cases had stable pseudo‐joints at the sacral site. The authors believe that the use of autologous living bone can help to better heal at the interface. A portion of the gluteus medius remains attached to the iliac bone graft, possibly providing abductor function. Among the nine cases, four cases did not heal after bone grafting, of which two cases had bone grafting absorption, one case died of brain metastasis 5 months after operation, and one case had deep infection and bone grafting absorption. The healing time was 5 to 7 months (average, 6.2 months). Titanium rod loosening and displacement occurred in two patients with nonunion of bone graft at 11 months and 24 months, respectively, without fracture of nail rod.

### *Limitation of the Study*

This study was a single‐center retrospective study. These cases were not randomized by treatment. The shortcomings were that the number of cases was small and the follow‐up time was short, so it was impossible to analyze the factors affecting prognosis.

In conclusion, resection of primary malignant bone tumors involving sacroiliac joint is difficult. The quality of resection margin and initial grade of tumors are the determinants of tumor prognosis. Combining short‐term metal stability with long‐term bone grafting is a good choice for pelvic ring reconstruction.
